# Expanded Yet Restricted: A Mini Review of the Soft Skills Literature

**DOI:** 10.3389/fpsyg.2020.02207

**Published:** 2020-09-04

**Authors:** Anna K. Touloumakos

**Affiliations:** ^1^Department of Education, Centre on Skills, Knowledge and Organisational Performance, University of Oxford, Oxford, United Kingdom; ^2^Department of Psychology, Panteion University of Social and Political Sciences, Athens, Greece

**Keywords:** soft skills, skill, soft skills conceptualization, soft skills development, curriculum design

## Abstract

There has been a progressively heightened preoccupation with soft skills among education stakeholders such as policymakers, educational psychologists, and researchers. Soft skill curricula have been considered these days and developed not only for graduates and as on-the-job training programs but also for students across all levels of education. However, different people mean different things when referring to soft skills. This review presents evidence to suggest that the use of the term “soft skills” has expanded to encompass a variety of qualities, traits, values, and attributes, as well as rather distinct constructs such as emotional labor and lookism. It is argued here that these infinite categories of things can be skills because soft skills research is primarily focused on what are the needs and requirements in the world of work. This approach is problematic because it assigns characteristics to soft skills, which in turn affect the design of the soft skills curricula. For example, soft skills are often construed as decontextualized behaviors, which can be acquired and transferred unproblematically. The paper proposes that an in-depth and embedded approach to studying soft skills should be pursued to reach a consensus on what they are and how to develop them because otherwise they will always be expanded before restricted (as they have become ambiguous) in their meaning and definition.

## Introduction

Suppose you are present in a communication encounter between two men, Joe and Martin. Joe looks upset and literally screams while recounting an incident that has happened to him:

*“Can you believe this?,”*, *Joe starts, “CAN YOU BELIEVE HIM? THE NERVE (.) he actually ended up ordering me “shut up, already, and do as I say!” (sounds infuriated) Joe is breathing heavily.*
Martin nods thoughtfully.
*(0.7) “As if he was in charge of me (.) as if he owned me*…*Where does he come off telling me what to do? Who does he think he is?” Joe continues.*
Martin nods again, pushing his glasses up the bridge of his nose.
(0.9) Joe seems lost in his thoughts.
*“I still can’t believe that this happened to me (.) It still makes me furious*…*”*
Martin’s nodding continues.
< *“Can you understand now why I acted the way I did?” Joe asks. “I was badly provoked* = = *What would you do if you were me?”* >
Martin responds with a nod.

Throughout Joe’s outburst, Martin keeps nodding. Nodding, in this instance, is an expressed form of active listening (Pasupathi et al., 1999; Browning and Waite, 2010). The behavior of listening as a unified whole, moreover, features most prevalently in the lists of communication skills encountered in the soft skills literature – educational, medical, management, policy, or other (see for example, Jain and Anjuman, 2013). However, is Martin’s behavior a communication skill as these lists inform us? Let us consider two alternative scenarios providing context for this exchange, which, hopefully, can help us decide.

In the first scenario, Martin is a clinical psychologist, and Joe is his client. Martin has been treating Joe for the past 2 years; he is, therefore, aware that Joe suffers from bipolar disorder and that, as part of this, he experiences, periodically, manic episodes, like the one he recounts in the aforementioned exchange taking place in a supermarket between him and a stranger. In this communication encounter, Martin’s nodding and listening are the expressive form of his active processing of the contents of this narrative. It can be argued that it realizes Martin’s relation to Joe (i.e., he is Joe’s therapist) and his intention to encourage him to let it out and that it is enacting Martin’s knowledge of Joe’s condition, the relevant symptoms, and the techniques to deal effectively with it. In line with this, it seems fair to suggest that Martin’s listening behavior is an effective communication strategy and that it can, therefore, be construed as a “communication skill.”

In the second scenario, Joe has just started working as an employee in his uncle’s business. He works along with other seven employees under a team leader (Jacob). The team reports to Martin, the line manager. Joe is difficult to work with and has been constantly reporting problems to Martin with either his team leader or other team members. In the above excerpt, he recounts a recent episode between him and his team leader, Jacob, when the former refuses to follow the agreed strategy during a negotiation meeting. The encounter between Joe and Martin is one of many within the past few weeks. In this instance, and contrary to what one might have expected from a line manager, Martin’s behavior toward Joe fails to articulate the sensible aim of reasoning with Joe and taking actions to ensure such fights come to an end. Martin’s behavior, therefore, seems to be guided by something different; a possible explanation could be that he fears his behavior might displease the boss, so he remains silent instead. If that is the case, could still the behavior of listening be construed as a “communication skill” within the context of this scenario?

These episodes aim to illustrate how meaningless it is to call *listening –* as a random sample of any of the behaviors commonly featured in the different soft skills lists *–* a communication skill, before having access to all contextual information that would allow making an informed judgment. However, as the review of the literature that follows highlights this is the norm conceptualization of soft skills: any behavior mobilized in a communication encounter can be taken out of context and find its place to a list of communication skills without any formal and scientific criterion for doing so. The review starts first with the norm approach in the conceptualization and use of the term “skill” – itself.

## Methods

### Sources and Search Strategy

The literature review for this mini-review article was undertaken at two separate points in time: in the first instance looking at the literature up to and including 2011 and later for years 2011–2020. During the first period (up to 2011), a review of the term soft skills formed part of the literature review undertaken as part of a doctorate thesis (Touloumakos, 2011). During this period, (a) keyword searches using the term “soft skills” (but also “soft skills” AND “characteristics,” “soft skills” AND “nature,” “soft skills” AND “development”) were conducted through the scientific databases: Google Scholar, Web of Science, and Scopus; (b) specific journals focusing on education, management, and the labor market were targeted and searched to meet the criteria of the doctoral research that looked at the difference between soft skills conceptualization in practice and in educational policy.

During this second period, the previous steps were reiterated to produce an up-to-date list of papers in which the term was used and defined. The author acknowledges that this article does not follow the methodology of a systematic review and that there is certainly scope for a thorough and systematic review on this topic in the future.

## Findings

### What Do We Mean by Skill?

The first known use of the term “skill” dates back in the 13th century (Merriam-Webster’s, 2019). Skill is considered as the “dexterity or coordination…in the execution of tasks” (typically of physical nature), as the “ability to use one’s knowledge effectively and readily in execution and performance,” and as “learned power of doing something competently.” The practical disposition of skill is acknowledged in these definitions. It is also highlighted in the work of Ryle (1949) and Polanyi (1962), according to who *skill* is construed as what knowledge sets in action (know-how and know-that, respectively), and, therefore, the two (knowledge and skill) are seen as “reciprocally constitutive” (Orlikowski, 2002). For the purposes of this paper, I adopt the view of *skill as what knowledge sets in action* (know-how).

## The Expansion of Skills: The Emergence of the Soft Skills Category

### Contributing Factors in the Expansion of the Term Skill

Over the years, the term skill has expanded considerably, to the point that its meaning became vague. In recent discourse, especially, it has taken on a range of meanings, and as a result, it refers, frequently, to “what is not skill” (Hart, 1978). Indicatively, the term often refers to attitudes, traits, volitions, and predispositions (*inter alia*, Payne, 2004; Clarke and Winch, 2006) and is sometimes confused, and even interchangeably used, with terms such as expertise and competence (Payne, 2000; Pring, 2004; Eraut and Hirsch, 2007). Its gradual expansion has meant (and is reflected in) the emergence of new skills categories and subcategories (indicatively: generic, soft, interpersonal, etc.). Key contributing factors toward such gradual expansion of the term skill are identified at three levels. The first is at the rhetorical level; the second is at the definitional level; and the third level is at the dispositional character of term itself within different scientific fields.

Focusing on the rhetorical level, in recent years, there has been a linguistic transition from terms such as “skilled work” and “skilled labor” to “skills.” Payne’s paper highlights this shift:

Whereas the Carr Report of 1958 (HMSO 1958: 10), for example, could still talk of “*skilled craftsmen*” [my emphasis] as being the “backbone of industry,” 40 years on, The Learning Age (Department for Education and Employment 1998: 65) was employing a much wider discourse of “basic skills,” “employability skills,” “technician skills,” “management skills,” and “key skills” (Payne, 2000, p. 353).

As is evident, the term “skill” (i.e., a noun) began to be used as an *independent concept* and replaced the use of the term as a *characteristic* referring to *people and professions* for example “skilled craftsmen,” “skilled labor,” and “skilled trades” (i.e., an adjective) in the policy rhetoric. The consistency of the use of “skill” in the literature reflects a tendency to turn the abstract notion of a “skilled craftsman” into something more concrete. In this transition, one can identify a reified conceptualization of skill, according to which “skill” is an entity – often a property of an individual (see Sfard, 1998; Clarke and Winch, 2006).

At the definitional level, the criteria of *what counts as skill* expanded considerably, which naturally meant the expansion of the term “skill” as well. Relevant here is the ongoing debate around which jobs should be placed on the high skills end of the spectrum (see Lloyd and Payne, 2008). In Marx’s work (1970), for example, distinguishing criteria for skilled job included the high wages and low levels of physical labor at the same time. More recent seminal theoretical work summarizes the criteria distinguishing “unskilled” and “skilled” jobs (Lloyd and Payne, 2008). The thinking behind such distinction is quite different from that of Marx. The authors discuss as an example (p. 1–2) the emergence of categories such as “emotional labor” (Hochschild, 1979, 1983) as a form of skilled labor “*requiring a range of quite complex and sophisticated abilities* (see Bolton, 2004, 2005; Korczynski, 2005).” The additional criteria for “what count as skill” in this work suggest a progressively ambiguous use of skill, which destined to term “skill” itself to ambiguity.

Third was the versatility of the term rendering it useful within the context of a range of scientific disciplines. Research on skills is rampant in the international literature, for example in cognitive studies – since many decades now – (Anderson et al., 1996, 1997), in education (Clarke and Winch, 2006; Eraut and Hirsch, 2007; Ritter et al., 2018), in policy-making (Wolf, 2004, 2011; Ewens, 2012; World Economic Forum [WEF], 2015; OECD, 2016; LINCS, 2020), in labor market studies (Meager, 2009; Kok, 2014), in management (Kantrowitz, 2005; Stevenson and Starkweather, 2010), or in medicine (Maguire and Pitceathly, 2002; Kurtz et al., 2005), to name a few. This evidence corroborates the multi-currency of “skill”,” which operationalizes *cognitive mechanisms*, *human capital (the worker)*, and *jobs and tasks*, depending on the discipline. It is because of this that we tend to speak of *people* and *work* in terms bundles of “skills” (Darrah, 1994). The problem is this seems hard to avoid considering that the “deeper one looks into any activity the more knowledge and skill one is likely to find” (Lloyd and Payne, 2009, p. 622 drawing from Attewell, 1990).

Taken together, this evidence suggests not only the “*conceptual equivocation*” (Payne, 2000) of the term as it is, but also the potentially perpetual emergence of new skills categories, a *“galaxy of “soft,” “generic,” “transferable,” “social,” and “interactional’ skills*” (p. 354).

### Soft Skills, Categories of Soft Skills, and Links Between Them

Soft skills were among the skills categories resulting from such expansion. While the emergence and use of the category of “soft skills” signified an important division between those skills that were cognitive and technical in nature – now frequently referred to as hard/technical skills – and those that were not, a unified view of the term in the literature has not been achieved. The genesis and use of the term are traced as far back as 1972 in training documents of the US Army (see Caudron, 1999; Moss and Tilly, 2001). Since then, the term has been expanded itself to comprise categories (in the various lists of soft skills) that include (but not exhaust to):

(a) *Qualities* (some of which one can see in the emotional intelligence literature) including adaptability, flexibility, responsibility, courtesy, integrity, professionalism, and effectiveness, and *values* such as trustworthiness and work ethic (see indicatively Wats and Wats, 2009; Touloumakos, 2011; Robles, 2012; Ballesteros-Sánchez et al., 2017);
(b) *Volitions*, *predispositions*, *attitudes like good attitude*, *willingness to learn*, *learning to learn other skills*, *hardworking*, *working under pressure, or uncertainty* (see indicatively Stasz, 2001; Stasz et al., 2007; Andrews and Higson, 2008; Cinque, 2017);
(c) *Problem solving*, *decision making, analytical thinking/thinking skills*, *creativity/innovation*, *manipulation of knowledge*, *critical judgment* (see indicatively Cimatti, 2016; Succi, 2019; Succi and Canovi, 2019; Thompson, 2019);
(d) *Leadership skills and managing skills* (see indicatively Crosbie, 2005; Lazarus, 2013; Ballesteros-Sánchez et al., 2017), as well as self*-awareness*, *managing oneself/coping skills* (see Cimatti, 2016; Cinque, 2017; Thompson, 2019);
(e) *Interpersonal savvy/skills*, *social skills*, and *team skills*, *effective, and productive interpersonal interactions* (see indicatively Kantrowitz, 2005; Bancino and Zevalkink, 2007; Succi and Canovi, 2019; Thompson, 2019);
(f) *Communication skills* (see indicatively Wats and Wats, 2009; Mitchell et al., 2010; Stevenson and Starkweather, 2010; Robles, 2012; Cinque, 2017) including elements of *negotiation*, *conflict resolution*, *persuasion skills*, *and diversity* (see, in addition, Bancino and Zevalkink, 2007; Majid et al., 2012; Cinque, 2017; Succi and Canovi, 2019) as well as *articulation work –* that is orchestrating simultaneous interactions with people, information, and technology (see Hampson and Junor, 2005; Hampson et al., 2009); but also going as far as.
(g) *Emotional labor* (originally from Hochschild, 1983), and even in some cases (in service jobs for example).
(h) *Aesthetics*, *professional appearance*, and “*lookism*” (see Nickson et al., 2005; Warhurst et al., 2009; Robles, 2012); finally,
(i) Other areas covered included *cognitive ability or processes* (see Cimatti, 2016; Ballesteros-Sánchez et al., 2017; Thompson, 2019), *ability to plan and achieve goals* (see Cimatti, 2016).

Next to the expansion of the categories comprising soft skills, the hierarchical relationships between the different categories of soft skills, as featured in the literature, added to its ambiguity. An example is the relationship between communication and interpersonal skills. In some places, the two terms are used as interchangeable; in some other cases, they are seen as two distinct categories forming alongside other categories of the construct of soft skills (Halfhill and Nielsen, 2007; Anju, 2009; Selvalakshmi, 2012; Jain and Anjuman, 2013). Finally, elsewhere, a hierarchical relationship exists between the two, namely the former is seen a part (a subcategory) of the latter (Rungapadiachy, 1999; Hayes, 2002; Harrigan et al., 2008). The simultaneous overlap, submerging, vicinity, and yet disparity of terms such as communication and interpersonal skills is just one of the many in the skills literature (cf. Kinnick and Parton, 2005, for discussion about overlap between communication and leadership). It becomes evident, accordingly, that these terms, much like the term soft skills has often become so stretched that their limits have become, in turn, vague. Their expansion meant actually that they became polysemous and, because of that, hard to grasp in a unified and organized way and therefore restricted in meaning and use.

## Discussion

This mini- review unveiled two important aspects in relation to the research and the conceptualization of soft skills. The first is that the rampant categories and lists of soft skills seem to be either the outcome of empirical work focusing on breaking down work activities (paraphrasing Lloyd and Payne, 2009) in addressing skills requirements, or recycled lists drawing from this work. This is the approach typically encountered in papers focusing on training graduates, training programes within organizations, and employers skills demands (for example Schulz, 2008; Constable and Touloumakos, 2009; Chamorro-Premuzic et al., 2010; Majid et al., 2012; Ballesteros-Sánchez et al., 2017; Succi and Canovi, 2019). This, however, can only be taken to be a veneer of an evidence-based approach to soft skills conceptualization, which is key for their understanding and development for two reasons:

(a) Because same categories mean different things and different categories mean same things to stakeholders (researchers, participants, policymakers), and
(b) Because the aim of researching skills requirements is very different to the aim of researching soft skills characteristics and their nature (soft skills conceptualization).

It is at the level of the conceptualization, characterization, and definition, therefore, that we need to pursue an evidence-based approach, so as to achieve a common language and avoid getting lost in translation in the use of the various soft skills terms.

The second aspect is that, in line with the way the literature features soft skills, they encompass such a wide and diverse range of categories (for example qualities, traits, values, predispositions, etc.) that makes it impossible to think about them as a coherent whole. Arguably, the warehousing approach of soft skills categories development, abstracts behaviors from the context of their enactment and call them skills. This approach, by definition, has ramifications for our understanding of soft skills characteristics, which in turn affects the thinking that underpins their development. For example, given that skills in line with this view are seen as actions toward tasks, it brings to the center *the person who acts* (Matteson et al., 2016) and, by extension, construes them as personal properties of a generic nature that can be first acquired and transferred uncomplicatedly across contexts (Touloumakos, 2011). Given that this (much like any other) conceptualization of soft skills affects the way we think about their development and their inclusion in education curricula, it is clear that a more inclusive, bottom—up and embedded view would provide a more pragmatic and meaningful alternative in their study.

## Author Contributions

This work has been undertaken in its entirety by AT.

## Conflict of Interest

The author declares that the research was conducted in the absence of any commercial or financial relationships that could be construed as a potential conflict of interest.
